# Extreme climatic events drive mammal irruptions: regression analysis of 100-year trends in desert rainfall and temperature

**DOI:** 10.1002/ece3.377

**Published:** 2012-09-21

**Authors:** Aaron C Greenville, Glenda M Wardle, Chris R Dickman

**Affiliations:** Desert Ecology Research Group, School of Biological Sciences, University of SydneySydney, New South Wales, 2006, Australia

**Keywords:** Climate change, dasyurids, extreme climate events, population dynamics Simpson Desert, rainfall, rodents, small mammals, temperature, thresholds

## Abstract

Extreme climatic events, such as flooding rains, extended decadal droughts and heat waves have been identified increasingly as important regulators of natural populations. Climate models predict that global warming will drive changes in rainfall and increase the frequency and severity of extreme events. Consequently, to anticipate how organisms will respond we need to document how changes in extremes of temperature and rainfall compare to trends in the mean values of these variables and over what spatial scales the patterns are consistent. Using the longest historical weather records available for central Australia – 100 years – and quantile regression methods, we investigate if extreme climate events have changed at similar rates to median events, if annual rainfall has increased in variability, and if the frequency of large rainfall events has increased over this period. Specifically, we compared local (individual weather stations) and regional (Simpson Desert) spatial scales, and quantified trends in median (50th quantile) and extreme weather values (5th, 10th, 90th, and 95th quantiles). We found that median and extreme annual minimum and maximum temperatures have increased at both spatial scales over the past century. Rainfall changes have been inconsistent across the Simpson Desert; individual weather stations showed increases in annual rainfall, increased frequency of large rainfall events or more prolonged droughts, depending on the location. In contrast to our prediction, we found no evidence that intra-annual rainfall had become more variable over time. Using long-term live-trapping records (22 years) of desert small mammals as a case study, we demonstrate that irruptive events are driven by extreme rainfalls (>95th quantile) and that increases in the magnitude and frequency of extreme rainfall events are likely to drive changes in the populations of these species through direct and indirect changes in predation pressure and wildfires.

## Introduction

With the global climate changing (IPCC 2007), it is becoming increasingly important to investigate how changes will be manifest at local and regional scales that are relevant to maintaining biodiversity (Chamaillé-Jammes et al. 2007; Peters et al. 2011). Beyond changes in average temperature and rainfall, extreme climate events such as flooding rain, droughts, or heat waves will alter in frequency and severity (CSIRO and Australian Bureau of Meteorology 2012). These infrequent but often catastrophic events may play key roles in regulating ecosystems, but understanding how event-driven dynamics govern diversity remains an elusive goal (Jentsch et al. 2011; Smith 2011a). We can expect fluctuations in climate to have direct effects on populations of organisms, but there are likely also to be many subtle or indirect effects, mediated through changes in resource availability or shifts in competitive or predatory relationships, that will further influence natural systems in complex ways (Brown et al. 1997; Smith et al. 1998; Epps et al. 2004).

Arid desert environments provide unique opportunities to explore the biotic effects of climatic extremes. Many deserts worldwide are characterized by extreme highs and lows in temperature, but the deserts in Australia are also distinctive for highly variable and unpredictable rainfalls (Van Etten 2009; Morton et al. 2011) that produce “boom” and “bust” dynamics in constituent biota (Letnic and Dickman 2010). Organisms have varied means of surviving these conditions, such as below-ground resting stages and life-history strategies that allow quick responses to extreme climate permutations (Noy-Meir 1973; Ward 2009; Morton et al. 2011). Temperatures in desert environments reflect, and in some regions exaggerate, the warming trend that has seen global temperatures rise 0.74°C since 1906 (IPCC 2007); fluctuations also are likely to be amplified in future (Ward 2009; CSIRO and Australian Bureau of Meteorology 2012). Increases in temperature in arid environments, and the resulting influence of this on wind patterns, can lead to changes in the rates of evaporation (Donohue et al. 2010) and to more subtle effects, such as reductions in animal body mass that have long-term consequences for the persistence of populations (Smith et al. 1998; Vincenzi et al. 2012). As global temperatures rise, the capacity of the atmosphere to hold moisture increases and circulation patterns also change (CSIRO and Australian Bureau of Meteorology 2007; IPCC 2007). Hence, changes in global rainfall may not reflect local rainfall changes and, in arid regions, may potentially result in further increases in rainfall variability. Increases and decreases in rainfall have been reported in arid regions of north America, central Australia, and Africa in recent decades (Allan et al. 1996; Brown et al. 1997; CSIRO and Australian Bureau of Meteorology 2007; Hoffman and Vogel 2008). For these reasons, deserts provide ideal templates to investigate how organisms respond to climate extremes over long periods. With improved understanding of the effects of climate fluctuations in the recent past we can then better anticipate how climate change may affect the biological diversity and function of these hypervariable systems in future.

Already high variability in rainfall and temperature in arid environments increases the challenges of detecting changes in long-term trends (Chamaillé-Jammes et al. 2007). A critical aspect of variability is changes in the maxima and minima for any parameter, as organisms will have to survive these extremes even if slight changes in mean values are not problematic. For example, temperatures exceeding 42°C cause death of flying foxes due to heat stress (Welbergen et al. 2008); hot conditions during drought can even cull populations of many specialist desert organisms (Low 2011 #1305; McKechnie, 2012 #1382; Fensham, 1999 #1385). In arid environments rainfall stimulates increases in primary productivity (Noy-Meir 1973; Morton et al. 2011), with extreme rainfall events (>90th quantile) driving interactions most strongly (Holmgren et al. 2006; Letnic and Dickman 2006; Smith 2011b; Popic and Wardle 2012). These pulse events are variable in time and space and often short lived (1–2 years), yet the resources they generate drive population “booms” in consumer organisms such as small mammals through bottom-up processes, provide prey for predators, and increase fuel loads that result in wildfires (Letnic and Dickman 2006; Pavey et al. 2008; Greenville et al. 2009; Letnic et al. 2011). Extreme weather events are predicted to increase in magnitude and frequency (IPCC 2007; CSIRO and Australian Bureau of Meteorology 2012) and thus may drive changes in arid systems disproportionately more than in mesic systems.

In this study, we collate long-term (100+ year) climate records from weather stations located on the periphery of the Simpson Desert, a vast arid region in central Australia, and examine trends in the climate over time. We compare trends at local (individual weather stations) and regional (Simpson Desert) scales, and focus in particular on changes in extreme climatic events which we define here as annual temperature or rainfall events in the 5th, 10th, 90th, and 95th quantiles. These quantiles thus represent extreme cold or dry (5th and 10th quantiles) and extreme hot or wet (90th and 95th quantiles) climate events. We also investigate if extreme climate events are changing at similar rates to median (50th quantile) events, and ask how one case study group of organisms – small mammals – has responded to climatic fluctuations over the last 22 years. Our climate-specific predictions are that:

1. annual temperatures, expressed as median and extreme values of maximum and minimum annual temperatures;
2. the magnitude of extreme annual rainfall events;
3. intra-annual rainfall variability; and
4. the frequency of extreme annual rainfall events

have all increased over time, but at different rates across local and regional scales. Conversely, we predict that (5) median annual rainfall will show an inconsistent pattern over time across local and regional scales. From previous work we assume that small mammals require some threshold in annual rainfall before their populations are able to irrupt, or “boom.” In addition, because extreme temperatures may affect mammal populations through heat stress, disruption of torpor, or other physiological processes, we predict that (6) thresholds for this case study group are extreme rainfall or minimum and maximum temperature events (>90th quantile). We use our findings to discuss the implications of change in extreme climate events for this arid system.

## Materials and methods

### Study region

**Figure 1:**
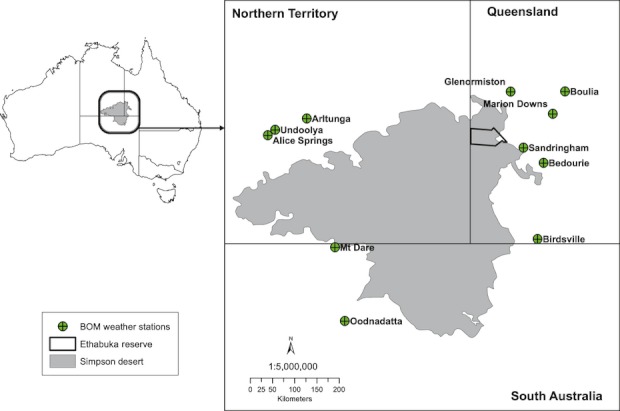
Location of study region; the Simpson Desert, Australia, in relation to the Bureau of Meteorology (BOM) weather stations. Ethabuka Reserve is the location of the live mammal trapping used in this study.

This study was carried out within the 200 and 400 mm rainfall isopleths in central Australia (Fig. 1). The Simpson Desert represents the major region in this rainfall band and occupies 170,000 square kilometers (Fig. 1); dune fields comprise 73% of this area, with smaller areas consisting of clay pans, rocky outcrops, and gibber flats (Shephard 1992). The sand dunes run parallel in a north-south direction aligned with the prevailing southerly wind. The dunes are up to 10 m high and spaced 0.6–1 km apart (Purdie 1984). Vegetation is predominantly grassland dominated by spinifex (*Triodia basedowii*) with interdune swales covered variously with small stands of gidgee trees (*Acacia georginae*), other woody *Acacia* shrubs, or mallee-form eucalypts; low-lying clay pans fill with water temporarily after heavy rain.

During summer, daily temperatures usually exceed 40º C and minima in winter often fall below 5°C (Purdie 1984). Rain falls mostly in summer, but heavy rains can occur locally or regionally throughout the year. Eleven weather stations closest to the desert (Fig. 1) have median annual rainfalls ranging from 153.1 to 216.2 mm collected over periods of 47–126 years (Bureau of Meteorology 2012).

### Temperature

Mean annual minimum and maximum temperature data (calendar year) were obtained from Boulia (1888–2011), Alice Springs (1878–2011), Birdsville (1954–2011), and Oodnadatta (1940–2011) weather stations (Bureau of Meteorology 2012), central Australia (Fig. 1). These stations were chosen because they have long-term (>50 years) temperature records and occur around the eastern and western edges of the Simpson Desert. Due to the remoteness of the study region and lack of any permanent settlements, no weather stations exist within the central region of the Simpson Desert.

To identify potential changes in the magnitude of extreme values of the above temperature variables over time (years), we used the median (50th quantile), two measures of the upper extreme values (90th and 95th quantiles), and two measures of the lower extreme values (5th and 10th quantiles) in analyses. Quantile regression was employed to test if the slopes of these quantiles (5th, 10th, 50th, 90th, and 95th) differed from zero. To estimate standard errors and *P*-values, we used a bootstrap method with 10,000 replacements as described in Koenker (1994). Quantile regressions were performed using the quantreg 4.76 package (Koenker 2011) in R 2.14.1 (R Development Core Team 2011).

Where significant nonzero slopes in annual temperatures were detected, confidence intervals were calculated for each parameter. Changes over time in extreme annual minimum and maximum temperatures (5th, 10th, 90th, and 95th quantiles) were each compared with changes in the median annual temperature (50th quantile), and significantly different changes at any time between an outlier quantile and the median were identified by nonoverlapping confidence intervals.

### Rainfall

Rainfall records (calendar year) within the 200 and 400 mm rainfall isopleths were obtained from 11 weather stations in the study region (Fig. 1): Arltunga (1901–2011), Glenormiston (1890–2011), Boulia (1886–2011), Marion Downs (1913–2011), Sandringham (1965–2011), Bedourie (1932–2011), Mt Dare (1950–2011), Oodnadatta (1892–2011), Alice Springs (1874–2011), Undoolya (1898–2011), and Birdsville (1892–2011) (Bureau of Meteorology 2012). These weather stations have the longest records for the Simpson Desert study region; without weather stations throughout the desert the records from these small towns and pastoral station localities provide the most comprehensive data available.

As for the temperature data, we used quantile regressions to investigate changes in the magnitude of extreme and median rainfall events over time (years). If changes were detected, we calculated confidence intervals for each parameter to determine if there was a different rate of change (slope) in the magnitude of extreme annual rainfall (5th, 10th, 90th, and 95th quantiles) compared with that of the median (50th quantile). Significant differences in rates of change occurred if the confidence intervals did not overlap.

To investigate if the frequency of extreme rainfall events increased over time, each extreme event (≥95th quantile) was given a consecutive number and the return time (years) until the next such event was calculated for 10 weather stations. A generalized linear mixed model (GLMM), with a log-link function and Poisson distribution was chosen to best model the return time, with *extreme event* as the fixed explanatory variable. *Weather station* was entered into the model as a random factor, with both intercept and slope permitted to vary. This allowed for local variation in annual rainfall and for relaxation of the assumption of equal slopes of return time for each weather station (Gelman and Hill 2007). GLMMs were performed using the lme4 package (Bates et al. 2011) in R 2.14.1 (R Development Core Team 2011).

We also calculated the coefficient of variation (CV) for monthly rainfall for each weather station to investigate temporal change in the variability of intra-annual rainfall. This was done by calculating the mean monthly rainfall for each year divided by its standard deviation. As above, quantile regression was used to investigate changes in the variability of annual rainfall in the median (50th quantile) and extreme ranges (5th, 10th, 90th, and 95th quantiles). This measure was used because there was only one weather station at each site and it was not appropriate to average over all weather stations due to high local variation.

### Small mammals

Live trapping of small mammals was carried out on Ethabuka Reserve, a 2140 km^2^ property located in the northeastern part of the Simpson Desert study region (Fig. 1). Small mammals were live trapped using pitfall traps (16 cm diameter, 60 cm deep), each equipped with a 5 m drift fence of aluminum flywire to increase trap efficiency (Friend et al. 1989). Pitfalls were arranged in a grid formation comprising six lines of six pitfall traps spaced 20 m apart to cover 1 ha. The top line of traps was positioned on a dune crest and the bottom line 100 m distant in the swale so that each grid sampled the topographic range of the dune field. The location of each grid was randomly chosen, with grids 0.5–2 km apart. Six grids were operated in 1990 and 12 for the remainder of the study (Dickman et al. 1999, 2001, 2011). Traps were opened for 3–6 nights every 2–4 months from 1990 to 2011. On each sampling occasion, captured animals were identified, weighed, individually marked, and sex determined as in Dickman et al. (1999, 2001). Captures were standardized per 100 trap nights (TN: trap nights = traps × nights opened) and averaged for each year.

To investigate whether small mammals responded to extreme rainfall and temperature events, the annual (calendar year) rainfall antecedent to capture and the annual minimum and maximum temperatures for captures that year were calculated from an on-site automatic weather station (Environdata, Warwick, Queensland). Missing values of rainfall were filled in from the nearest weather station at Sandringham, 63 km away (*r* = 0.81, *P* < 0.001), and missing values of temperature were filled from Boulia, 173 km away (*r* = 0.56, *P* = 0.02). The 1-year lag was used to account for the time required for small mammals to respond, via immigration and breeding, to a rainfall event.

We divided mammal captures from the 22 years of live trapping (397,476 trap nights) into rodents and dasyurids as these groups respond differently to rainfall and have different physiological strategies in response to food shortages (Dickman et al. 1999, 2001; Geiser 2004; Haythornthwaite and Dickman 2006). Rodents included *Pseudomys hermannsburgensis* (sandy inland mouse; 4208 captures), *Notomys alexis* (spinifex hopping mouse; 2731 captures), *P. desertor* (desert mouse; 858 captures), *Rattus villosissimus* (long-haired rat; 386 captures), and *Mus musculus* (house mouse; 306 captures). Dasyurids included *Dasycercus blythi* (brush-tailed mulgara; 438 captures), *Ningaui ridei* (wongai ningaui; 501 captures), *Sminthopsis youngsoni* (lesser hairy-footed dunnart; 1301 captures), *S. hirtipes* (hairy-footed dunnart; 83 captures), *S. macroura* (striped-faced dunnart; 4 captures), and *S. crassicaudata* (fat-tailed dunnart; 10 captures).

Piecewise regression was used to identify any thresholds in annual rainfall and temperature at which rodents and dasyurids responded. A threshold relationship was predicted as extreme rains and temperatures (>90th quantile) are more likely to drive small mammal populations in arid Australia than moderate rains or temperatures; linear relationships probably do not occur (Letnic and Dickman 2010). Piecewise regressions use two or more lines, joined at a break point, that can be used to identify thresholds (Toms and Lesperance 2003). We used piecewise regressions with one knot, or break point, that could occur at any rainfall or temperature value. The break point was estimated from likelihood-ratio statistics (Toms and Lesperance 2003). Confidence intervals were estimated from 1000 bootstrap samples. Piecewise regressions were performed using SiZer 0.1-4 (Sonderegger 2011) in R 2.14.1 (R Development Core Team 2011).

## Results

### Temperature

**Figure 2:**
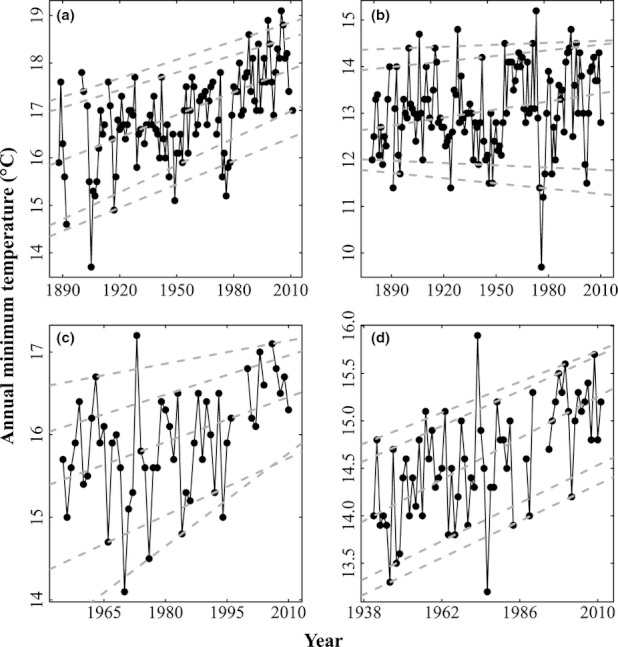
Mean minimum annual temperatures (°C) from (a) Boulia, (b) Alice Springs, (c) Birdsville, and (d) Oodnadatta weather stations, Simpson Desert, central Australia. Broken gray lines show 95th, 90th, 50th, 10th, and 5th quantiles.

**Figure 3:**
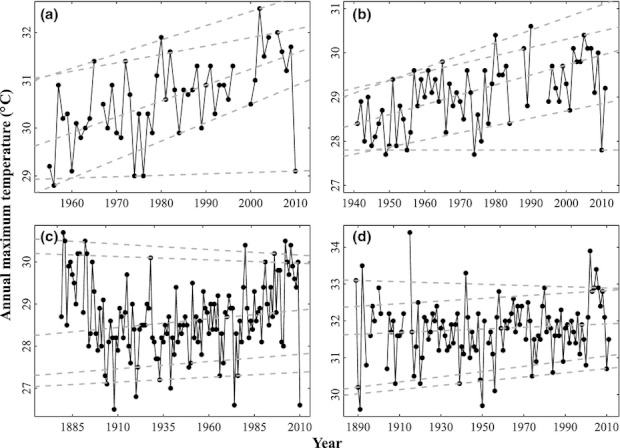
Mean maximum annual temperatures (°C) from (a) Birdsville, (b) Oodnadatta, (c) Alice Springs, and (d) Boulia weather stations, Simpson Desert, central Australia. Broken gray lines show 95th, 90th, 50th, 10th, and 5th quantiles.

Mean minimum annual temperatures showed significant warming over the period of study at three weather stations (Boulia, Birdsville, and Oodnadatta) in most quantiles, with median temperatures (50th quantile) increasing by up to almost 2°C (Fig. 2a–d). The median temperature increased also at the fourth weather station, Alice Springs, but no clear changes were apparent in extreme minimum temperatures (Table 1, Fig. 2b). Mean maximum annual temperatures showed significant warming of >1°C over the study period at two weather stations (Birdsville and Oodnadatta), especially in the 10th, 50th, 90th, and 95th quantiles (Table 2, Fig. 3a,b). No significant trends were apparent at Alice Springs and Boulia on the northwestern and northeastern fringes, respectively, of the Simpson Desert (Table 2, Fig. 3c,d). There was no difference in the rate of increase in either the extreme minimum or maximum annual temperatures compared with the rate of increase in median temperatures (Tables 1 and 2).

**Table 1:** Quantile regression results for minimum temperature change over time (years), Simpson Desert, central Australia

Weather Station Quantile	Estimate	CI	*t*-value	*P*
Boulia				
0.05	0.02	0.01–0.03	1.75	0.08
0.1	0.02	0.008–0.03	3.49	0.001*
0.5	0.02	0.01–0.02	6.07	<<0.001*
0.9	0.01	0.007–0.02	2.94	0.004*
0.95	0.01	0.009–0.02	3.50	0.001*
Alice Springs				
0.05	−0.004	−0.007–0.006	−0.95	0.34
0.1	−0.002	−0.005–0.003	−0.46	0.65
0.5	0.006	−0.00002–0.01	1.83	0.07
0.9	0.004	0.0004–0.01	1.31	0.19
0.95	0.001	−0.003–0.01	0.39	0.70
Birdsville				
0.05	0.04	−0.03–0.06	1.98	0.24
0.1	0.02	−0.01–0.06	1.18	0.24
0.5	0.02	0.01–0.03	3.20	0.002*
0.9	0.02	0.006–0.02	2.14	0.04*
0.95	0.009	−0.007–0.03	1.06	0.29
Oodnadatta				
0.05	0.02	0.006–0.04	2.31	0.02*
0.1	0.02	0.007–0.02	2.77	0.007*
0.5	0.02	0.015–0.02	6.21	<<0.001*
0.9	0.01	0.007–0.02	4.81	<<0.001*
0.95	0.01	0.01–0.03	2.43	0.02*

In each row a significant *P*-value indicated by * implies that there is a nonzero slope for that quantile. For each weather station (Boulia, Alice Springs, Birdsville, Oodnadatta) the slope estimate for each of the extreme quantiles (0.5, 0.1, 0.9, and 0.95) was compared with the median quantile (0.5). Overlaps in the confidence intervals (CI) indicated similar rates of change for all comparisons.

**Table 2:** Quantile regression results for maximum temperature change over time (years), Simpson Desert, central Australia

Weather station Quantile	Estimate	CI	*t*-value	*P*
Boulia				
0.05	0.01	0.003–0.01	1.62	0.11
0.1	0.007	0.002–0.01	1.65	0.10
0.5	0.003	−0.003–0.005	0.98	0.23
0.9	0.005	−0.003–0.007	0.99	0.32
0.95	−0.002	−0.005–0.004	−0.31	0.76
Alice Springs				
0.05	0.002	−0.02–0.02	0.28	0.78
0.1	0.003	−0.01–0.01	0.48	0.63
0.5	0.004	−0.001–0.009	1.29	0.20
0.9	−0.002	−0.004–0.001	−0.61	0.54
0.95	−0.003	−0.004–0.002	−1.07	0.29
Birdsville				
0.05	0.003	−0.004–0.06	0.12	0.90
0.1	0.04	0.01–0.06	1.95	0.06
0.5	0.03	0.02–0.04	4.30	<<0.001*
0.9	0.02	0.01–0.05	1.55	0.12
0.95	0.03	0.006–0.04	2.73	0.01*
Oodnadatta				
0.05	0	−0.004–0.03	0	1
0.1	0.01	−0.001–0.02	1.97	0.05*
0.5	0.02	0.01–0.03	3.98	<<0.001*
0.9	0.02	0.01–0.03	2.99	0.004*
0.95	0.03	−0.002–0.04	3.50	<<0.001*

In each row a significant *P*-value indicated by * implies that there is a nonzero slope for that quantile. For each weather station (Boulia, Alice Springs, Birdsville, Oodnadatta) the slope estimate for each of the extreme quantiles (0.5, 0.1, 0.9, and 0.95) was compared with the median quantile (0.5). Overlaps in the confidence intervals (CI) indicated similar rates of change for all comparisons.

### Rainfall

**Figure 4:**
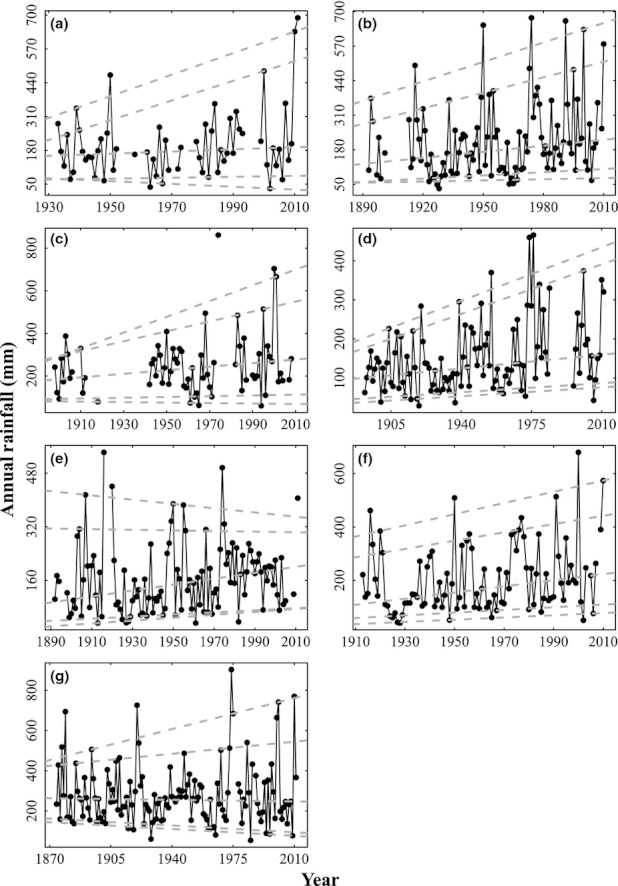
Annual rainfall (mm) recorded each year for (a) Bedourie, (b) Glenormiston, (c) Undoolya, (d) Oodnadatta, (e) Birdsville, (f) Marion Downs, and (g) Alice Springs weather stations, Simpson Desert, central Australia. Gray lines show 95th, 90th, 50th, 10th, and 5th quantile. The 90 and 95th quantiles represent boom years, 50th is the median, and 10th and 5th represent bust years.

The magnitude and direction of change in annual rainfall varied among the long-term weather stations in central Australia, with some indication of both local and regional trends. Four weather stations (Bedourie, Glenormiston, Undoolya, and Oodnadatta) showed significant increases in the magnitude of extreme rainfall events (90th and 95th quantiles; Fig. 4a–d), with peak rainfalls roughly doubling in size over the study period; two weather stations (Birdsville and Marion Downs) showed increases in median rainfall (50th quantile; Table 3, Fig. 4e,f). Two weather stations (Birdsville and Alice Springs) showed significant changes in rainfall during extreme dry years (5th and 10th quantiles), but the direction of these changes was inconsistent (Table 3; Fig. 4e,g). Four weather stations (Boulia, Sandringham, Arltunga, and Mt. Dare) showed no significant relationship (Table 3, Fig. S1a–d). In general, extreme annual rainfall events increased in magnitude around the Simpson Desert, whereas increases in the magnitude of median rainfall events were restricted to the eastern desert regions. In the west of the Simpson Desert extremely dry years became drier over the study period, whereas the reverse pattern occurred in the east.

**Table 3:** Quantile regression results for annual rainfall changes over time (years) for each weather station, Simpson Desert, central Australia

Weather Station Quantile	Estimate	CI	*t*-value	*P*
Bedourie				
0.05	−0.54	−0.13–1.89	−0.77	0.44
0.1	0.17	−0.83–1.84	0.28	0.78
0.5	0.41	−0.17–1.26	0.65	0.52
0.9	3.78	0.68–5.05	1.83	0.07
0.95	**4.15**	2.10–5.10	2.03	0.04*
Glenormiston				
0.05	0.15	−0.17–0.97	0.41	0.68
0.1	0.40	−0.18–1.10	1.32	0.19
0.5	0.76	−0.12–1.64	1.71	0.09
0.9	1.95	0.36–3.10	1.79	0.07
0.95	**2.44**	1.06–3.68	2.05	0.04*
Marion Downs				
0.05	0.42	−0.15–0.73	1.11	0.27
0.1	0.50	−0.003–1.17	1.18	0.24
0.5	1.15	0.62–1.93	2.37	0.02*
0.9	1.55	−0.08–3.72	1.11	0.27
0.95	2.10	0.10–3.30	1.41	0.16
Boulia				
0.05	0.19	−0.90–0.35	0.53	0.59
0.1	−0.16	−0.61–0.52	−0.53	0.59
0.5	0.79	−0.69–1.04	1.52	0.13
0.9	−1.77	−2.37–1.51	−1.25	0.21
0.95	0.07	−2.45–3.26	0.04	0.97
Birdsville				
0.05	0.43	−0.15–0.59	1.97	0.05*
0.1	0.31	0.15–0.64	1.74	0.09
0.5	0.89	0.31–1.39	2.48	0.01*
0.9	−0.09	−2.24–1.71	−0.07	0.94
0.95	−0.63	−3.34–1.48	−0.42	0.67
Sandringham				
0.05	−0.79	−2.67–2.24	−0.42	0.67
0.1	−1.39	−2.41–2.09	−0.92	0.36
0.5	−1.95	−3.81–1.70	−1.18	0.24
0.9	1.24	−6.90–3.39	0.38	0.70
0.95	0.56	−7.36–26.32	0.16	0.87
Arltunga				
0.05	−0.14	−5.17–1.00	−0.31	0.75
0.1	−0.24	−1.32–0.80	−0.57	0.57
0.5	0.10	−0.72–1.32	0.15	0.88
0.9	1.67	0.47–4.55	0.95	0.34
0.95	1.70	0.55–5.54	0.80	0.42
Undoolya				
0.05	−0.11	−0.41–2.43	−0.24	0.81
0.1	0.18	−0.33–1.63	0.37	0.71
0.5	0.84	−0.89–0.98	1.38	0.17
0.9	2.32	0.83–3.75	1.77	0.08
0.95	**3.70**	1.69–4.17	2.45	0.02*
Alice Springs				
0.05	−0.48	−0.86 to −0.32	−2.41	0.02*
0.1	−0.48	−6.66 to −0.01	−1.96	0.05*
0.5	−0.11	−0.31–0.69	−0.36	0.72
0.9	0.86	−0.70–2.66	0.72	0.47
0.95	2.20	0.50–2.51	1.84	0.07
Oodnadatta				
0.05	0.32	−0.05–0.74	1.27	0.21
0.1	0.33	−0.09–0.56	1.50	0.14
0.5	0.50	0.18–1.00	1.91	0.06
0.9	**1.81**	1.16–2.20	3.83	<<0.001*
0.95	1.96	0.97–3.70	2.10	0.04*
Mt Dare				
0.05	0.58	−0.98–1.71	0.83	0.41
0.1	0.51	−0.36–1.66	0.74	0.46
0.5	0.75	−0.77–1.56	0.94	0.35
0.9	−2.33	−8.39–3.68	−0.71	0.48
0.95	−2.27	−8.80–10.63	−0.51	0.61

The estimates in bold indicate that the slope does not overlap the confidence interval (CI) for the median slope (0.5 quantile). Significant *P*-value indicated by *.

The rate of change (increase) in the magnitude of extreme rainfall events (90th and 95th quantiles) was greater than the median rate of change at four weather stations (Bedourie, Glenormiston, Undoolya, and Oodnadatta; Table 3), indicating a desert-wide increase in the size of extremely heavy rainfall events over the last century.

### Extreme rainfall return intervals

There was a marked decrease in the number of years between extreme rainfall events (>95th quantile) (GLMM estimate: −0.54, SE = 0.21, *z*-value = −2.54, *P* = 0.01) across the Simpson Desert, indicating that such events have become more frequent over time.

### Rainfall variability

The coefficient of variation in rainfall decreased over the period of study at three weather stations, Bedourie, Glenormiston, and Boulia (Table 4). Variability in rainfall decreased in magnitude during extreme dry years at the first two of these stations and showed a decline in the median rainfall at Boulia. All three weather stations are on the eastern edge of the Simpson Desert, suggesting that this trend is not consistent across central Australia.

**Table 4:** Quantile regression results for intra-annual rainfall coefficient of variation for weather stations in the Simpson Desert, central Australia

Weather Station Quantile	Estimate	SE	*t*-value	*P*
Bedourie				
0.05	−0.009	0.002	−4.62	<<0.001*
0.1	−0.008	0.002	−3.37	0.001*
0.5	−0.002	0.004	−0.49	0.63
0.9	0.003	0.004	0.63	0.53
0.95	0.007	0.004	1.75	0.09
Glenormiston				
0.05	−0.002	0.002	−0.1	0.33
0.1	−0.003	0.001	−2.39	0.02*
0.5	−0.004	0.002	−1.81	0.07
0.9	−0.001	0.004	−0.31	0.76
0.95	−0.001	0.003	−0.41	0.68
Marion Downs				
0.05	−0.0004	0.002	−0.17	0.87
0.1	−0.003	0.003	−0.96	0.34
0.5	−0.0007	0.002	−0.08	0.93
0.9	−0.001	0.005	−0.19	0.85
0.95	0.003	0.005	0.64	0.52
Boulia				
0.05	−0.002	0.002	−1.1	0.28
0.1	−0.0004	0.002	−0.29	0.78
0.5	−0.003	0.001	−2.83	0.005*
0.9	−0.0002	0.002	−0.07	0.95
0.95	0.0006	0.002	0.31	0.76
Birdsville				
0.05	−0.0002	0.002	−0.1	0.92
0.1	0.0008	0.002	0.51	0.61
0.5	0.0003	0.001	0.25	0.81
0.9	−0.005	0.004	−1.23	0.22
0.95	−0.006	0.004	−1.8	0.08
Sandringham				
0.05	−0.00002	0.008	−0.002	0.99
0.1	−0.009	0.008	−1.11	0.27
0.5	0.007	0.008	0.93	0.36
0.9	−0.008	0.01	−0.79	0.44
0.95	0.001	0.009	0.12	0.90
Arltunga				
0.05	−0.0009	0.002	−0.52	0.61
0.1	−0.002	0.002	−0.85	0.40
0.5	−0.0007	0.002	−0.37	0.71
0.9	−0.0006	0.002	−0.26	0.80
0.95	−0.0003	0.003	−0.09	0.93
Undoolya				
0.05	0.0003	0.003	0.09	0.93
0.1	0.002	0.002	1.29	0.20
0.5	0.003	0.002	1.28	0.21
0.9	0.0002	0.004	0.06	0.95
0.95	−0.004	0.006	−0.67	0.51
Alice Springs				
0.05	−0.0005	0.0009	−0.58	0.56
0.1	−0.0008	0.0009	−0.84	0.40
0.5	0	0.001	−0.001	1
0.9	0.003	0.002	1.60	0.11
Oodnadatta				
0.95	0.003	0.003	0.98	0.33
0.05	−0.003	0.002	−1.06	0.29
0.1	−0.002	0.002	−0.79	0.43
0.5	0.0003	0.001	0.23	0.82
0.9	−0.002	0.005	−0.38	0.70
0.95	0.00008	0.006	0.01	0.99
Mt Dare				
0.05	−0.0005	0.006	−0.09	0.93
0.1	0.003	0.004	0.86	0.39
0.5	−0.002	0.004	−0.49	0.63
0.9	−0.008	0.01	−0.72	0.47
0.95	0.003	0.01	0.20	0.84

Significant *P*-value indicated by *.

### Small mammals

**Figure 5:**
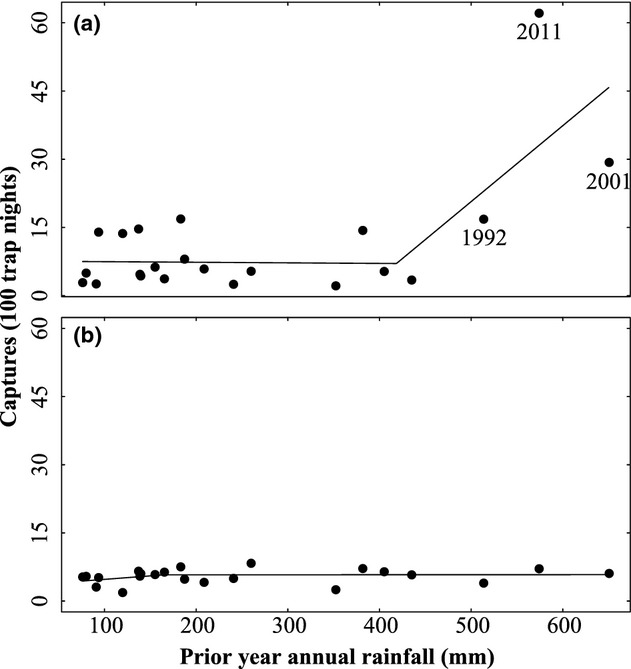
The response of (a) rodent and (b) dasyurid captures to rainfall in the preceding year. Lines depict predicted values from piecewise regression. Rodent captures show a response to rainfall only after a threshold of 418 mm (∼0.95 quantile) is reached. Labels on (a) indicate year of irruption. No relationship between rainfall and dasyurids was found. Small mammal captures standardized per 100 trap nights (TN) and averaged per year (*n* = 22 years).

Rodent captures showed a strongly positive response to rainfall in the preceding year at a threshold of 418 mm (CI: 240–506 mm; Table 5; Fig. 5a). This represents an extreme annual rainfall event in the ∼95th quantile on Ethabuka Reserve. In contrast, dasyurid captures showed no response to annual rainfall from the preceding year (Table 5; Fig. 5b). There was no significant relationship for rodents or dasyurids and minimum or maximum temperatures (Table 6).

**Table 5:** Piecewise regression results for the response of small mammal captures to annual rainfall from the preceding year, Ethabuka Reserve, Simpson Desert, central Australia

	Estimate	SE	*t*-value	*P*
Rodents				
Intercept	7.60	4.42	1.72	0.10
Line 1	−0.001	0.02	−0.07	0.95
Line 2	0.17	0.05	3.11	0.006*
Dasyurids				
Intercept	3.24	1.77	1.83	0.08
Line 1	0.02	0.01	1.19	0.25
Line 2	−0.02	0.01	−1.06	0.30

Small mammal captures standardized per 100 trap nights (TN) and averaged per year (*n* = 22 years). Significant *P*-value indicated by *.

**Table 6:** Piecewise regression results for the response of small mammal captures to annual minimum and maximum temperatures, Ethabuka Reserve, Simpson Desert, central Australia

	Estimate	SE	*t*-value	*P*
Minimum temperature				
Rodents				
Intercept	9.81	40.68	0.24	0.81
Line 1	−0.04	2.94	−0.01	0.99
Line 2	1.87	4.60	0.41	0.69
Dasyurids				
Intercept	−38.21	25.42	−1.50	0.15
Line 1	3.78	2.19	1.72	0.10
Line 2	−3.91	2.26	−1.73	0.10
Maximum temperature				
Rodents				
Intercept	88.49	83.70	1.06	0.30
Line 1	−2.41	2.60	−0.93	0.37
Line 2	7.10	9.07	0.78	0.44
Dasyurids				
Intercept	−3.70	11.31	−0.33	0.75
Line 1	0.28	0.35	0.81	0.43
Line 2	−0.24	0.71	−0.34	0.74

Small mammal captures standardized per 100 trap nights (TN) and averaged per year (*n* = 22 years). Significant *P*-value indicated by *.

## Discussion

We predicted that recent global warming would be reflected by increases in temperatures on local (individual weather stations) and regional (Simpson Desert) scales, for both median and extreme temperature values. In contrast, we predicted increases in the size and frequency of extreme rainfall events over time in the Simpson Desert, but expected median rainfall to vary inconsistently at both spatial scales. In general, temperatures increased largely as predicted whereas trends in rainfall showed local and regional variation; the magnitude of heavy rainfall events increased at four weather stations on the eastern and western fringes of the Simpson Desert, and the frequency of extreme rainfall events also increased. Small mammals showed differing responses to rainfall; rodent populations irrupted only after an extreme rainfall event (>95th quantile) whereas dasyurids did not. There was no response by small mammal populations to temperature. Here, we address each climate variable in turn and then, using small mammals as a case study, we discuss the likely responses of desert organisms to climate change.

### Temperature

Our results confirm that there has been significant warming in the Simpson Desert region over the last century. Mean annual night temperatures have increased, with 6 of the 12 hottest nights occurring since 1970 (90–95th quantiles) and the coolest nights also warming over the study period (10th quantile; Fig. 2a,c,d). This warming has occurred locally and regionally, suggesting that increases in global temperatures manifest at both scales. These results are consistent with previous studies in Australia (Hughes 2003, 2011; Trewin and Vermont 2010) and globally (IPCC 2007; Tabari and Hosseinzadeh Talaee 2011). We found similar rates of change in both median and extreme annual temperatures, suggesting that increases in global temperatures have affected median and extreme temperatures equally.

Global temperatures are predicted to increase by 0.6–4°C by the end of the 21st century and Australian temperatures to increase by 1–5°C by 2070, depending on emission scenarios (CSIRO and Australian Bureau of Meteorology 2007; IPCC 2007). The northwestern and central regions of Australia are predicted to show the greatest warming in both minimum and maximum temperatures (CSIRO and Australian Bureau of Meteorology 2007). Our results showing changes in both the median and extreme temperatures are consistent with these predictions.

### Rainfall

There was variability in long-term annual rainfall trends at local and regional scales. The Bedourie, Undoolya, Oodnadatta, and Glenormiston weather stations showed significant increases in 95th quantile rainfall events (Table 3), suggesting that the magnitudes of these large rainfalls (>300 mm) have increased in recent decades. Marked increases in median rainfalls were found for the Marion Downs and Birdsville weather stations, and increases in rainfall during extremely dry years (5th quantile) occurred at Birdsville. In contrast, rainfall at Alice Springs decreased during extremely dry years (Table 3). Rainfall trends in Australia show strong spatial variability (CSIRO and Australian Bureau of Meteorology 2007). At the continental scale there has been a nonsignificant increase in rainfall (Hennessy et al. 1999), but regional patterns are more clear (Hennessy et al. 1999; Hughes 2003). Since 1950, annual rainfall has increased in the north-west of the continent and decreased in the east; in central arid regions annual rainfall generally has increased (CSIRO and Australian Bureau of Meteorology 2007). We found that annual rainfalls have increased during extremely wet years, whereas increases in the magnitude of median rainfall events were restricted to eastern parts of the Simpson Desert, with some local variability. In the west of the Simpson Desert extremely dry years received reduced annual rainfall, but in the east there were increases. Higher rainfall has been linked to increases in both heavy rainfall events and number of rain days (Hennessy et al. 1999; Hughes 2003; CSIRO and Australian Bureau of Meteorology 2007), which is consistent with increases in rainfall in the 95th quantile in our study.

As global temperatures rise, the moisture-holding capacity of the atmosphere increases and circulation patterns change, with resultant impacts on global rainfall (CSIRO and Australian Bureau of Meteorology 2007; IPCC 2007). By the end of the 21st century rainfall is predicted to increase at high latitudes and decrease in subtropical regions (IPCC 2007). Discrepancies in rainfall predictions for Australia by the end of the 21st century arise largely from the use of different approaches to determine the base level of rainfall (Hughes 2003; CSIRO and Australian Bureau of Meteorology 2007). Some models predict an increase in rainfall from tropical northern Australia to parts of the arid interior, whereas others predict a general decline in rainfall across most of Australia (CSIRO and Australian Bureau of Meteorology 2007). We found that changes in median rainfall over the last century differed across the regional study area. Two weather stations showed a significant increase in annual median rainfall, whereas the other stations showed no pattern (Table 3). The intensity of extreme rainfall events is predicted to increase across most of Australia (CSIRO and Australian Bureau of Meteorology 2007) and globally (IPCC 2007). Rainfall patterns in Australia are influenced by the El Niño Southern Oscillation, which is predicted to intensify due to climate change (IPCC 2007). We found increases in 95th quantile rainfall in the Simpson Desert to have occurred at significantly greater rates than the median; this is consistent with suggestions that past changes in extreme rainfall events will continue into the 21st century, although considerable spatial variability is likely to occur.

In general, the time between extremely large rainfall events (>95th quantile) decreased over time in the Simpson Desert, suggesting that the frequency of extreme rainfall events is increasing. This is consistent with the predictions of global climate change models (IPCC 2007) and with predictions for the future Australian climate (CSIRO and Australian Bureau of Meteorology 2007, 2012).

We predicted that annual rainfall would become more variable due to the impacts of climate change in the Simpson Desert, but found no support for this at either local or regional scales. Instead, we found a decrease in rainfall variability during extremely dry years – implying longer droughts – at two weather stations and for median rainfall at another weather station. All three weather stations are on the eastern edge of the Simpson Desert, suggesting that this trend is not consistent across the study region.

### Case study: small mammals

The two groups of small mammals in this study showed contrasting responses to annual rainfall and no response to annual minimum and maximum temperatures. Captures of rodents increased only after extreme rainfall years (∼95th quantile, or >400 mm rain), whereas dasyurid captures showed no response. In arid regions, increases in small mammal populations after rainfall are facilitated by increases in the availability of food (Dickman et al. 1999, 2011; Letnic et al. 2005, 2011), with rodents exhibiting the strongest responses (Dickman et al. 1999; Lima and Jaksic 1999; Lima et al. 2002, 2008; Pavey et al. 2008). Dasyurids do not appear to be limited by rainfall, but by other factors such as vegetation cover and life-history constraints (Dickman et al. 2001). Most species breed once in the Austral winter–spring and thus have limited opportunity to respond to infrequent, heavy rainfall events (Dickman et al. 2001).

With increases in the magnitude and frequency of extreme rainfall events recorded in at least some local areas of the Simpson Desert in recent decades, associated rodent irruptions are likely to have taken place. Historical records confirm that rodents have frequently irrupted following years receiving 95th quantile rainfall (e.g., Cleland 1918; Newsome and Corbett 1975; Watts and Aslin 1981), and our 22-year database suggests further that the size of irruptions is related to the amount of rain received (Fig. 5a). If the trends that we have identified continue, rodent irruptions will likely occur more frequently in future. It is possible also that populations of the constituent rodent species will achieve greater densities during outbreaks; very large population increases may be dampened by intraspecific competition or other density-dependent interactions (Lima and Jaksic 1999; Lima et al. 2008), but these are yet to be demonstrated in this study region (Dickman et al. 2010).

### Implications of climate change

Continuing increases in the frequency and magnitude of extreme rainfall events will likely have several important ecological consequences for biodiversity in inland Australia. We draw here on our observations of small mammals to provide focus, but acknowledge that most organisms will be affected directly and indirectly by temperature and rainfall shifts.

First, whereas irruptions of native rodents can be expected to increase, new species also may be able to establish during resource-rich boom conditions. Introduced rodent species such as the house mouse (*Mus musculus*), which is largely absent from the study region in dry times, may become more common (Letnic and Dickman 2010). Second, novel predators also may use boom conditions to become established. There is considerable evidence that feral cat (*Felis catus*) and red fox (*Vulpes vulpes*) populations can increase in the wake of extreme rainfall events, either by elevating their breeding *in situ* or by moving in from more mesic environments near the study region in response to elevated populations of rodent prey (Letnic et al. 2005; Letnic and Dickman 2006; Pavey et al. 2008). Top-down processes then may become stronger and more pervasive, reducing small mammal populations to very low levels during droughts (Letnic et al. 2011). Third, other novel interactions may develop; these include extended floral–pollinator networks that can be supported during periods of high productivity, and hyperpredation of seeds, green vegetation, and invertebrates that can arise when rodent populations are high (Brown et al. 1997; Bellard et al. 2012; Popic and Wardle 2012). Finally, the risk of wildfires increases after two consecutive years of rainfall that exceeds the 91st quantile in the study region (Letnic and Dickman 2006; Greenville et al. 2009). An increase in the magnitude and frequency of extreme rainfall events thus may promote higher fuel loads, increase fire risk through increased temperatures and evaporation, and decrease the fire return interval. Increased incidence of wildfires may favor species that do not require high vegetation cover such as some agamid species, but disadvantage many others that need dense cover (Haythornthwaite and Dickman 2006; Pianka and Goodyear 2012). As vegetation also provides a food resource for invertebrates and protection from predation, its removal by fire could exacerbate the negative effects of predators on small mammal populations (Letnic and Dickman 2006).

If the identified climatic trends continue, as predicted, rodents will probably experience exaggerated boom and bust cycles that will be characterized by large irruptions and deep and prolonged troughs. These dynamics will likely create conditions that are conducive to stochastic population extinctions at local or regional levels (Xu et al. 2006), and also allow suites of novel ecological interactions to develop that will influence small mammal assemblages in different ways. We conclude that these novel conditions will complicate attempts to conserve biodiversity in arid environments, but suggest also that long-term datasets and analyses of the biotic effects of extreme events will provide important insights into this task.

## Supporting Information

**Figure S1.** Annual rainfall (mm) recorded each year for a) Boulia, b) Sandringham, c) Arltunga, and d) Mt. Dare weather stations, Simpson Desert, central Australia. Grey lines show 95th, 90th, 50th, 10th and 5th quantile. 90 and 95th quantiles represent boom years, 50th is the median and 10th and 5th represent bust years.
