# Supplementary figures and images for: Extreme climatic events drive mammal irruptions: regression analysis of 100-year trends in desert rainfall and temperature
Source: Ecol Evol. 2012 Sep 21;2(11):2645–58. doi: 10.1002/ece3.377 (PMC3501619; doi:10.1002/ece3.377)

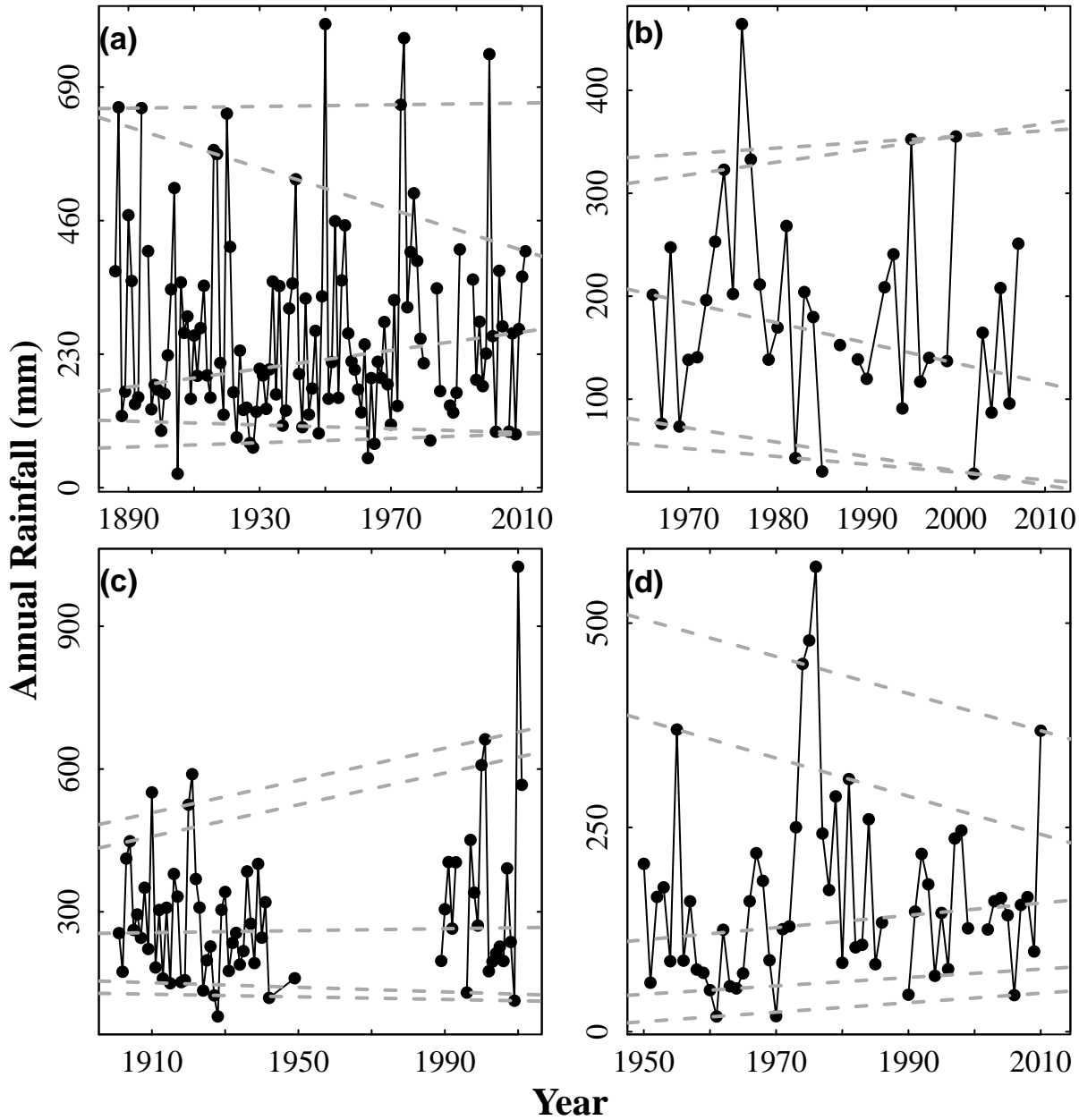

Supplement: Supplementary file 1 [file ece30002-2645-SD1.pdf]

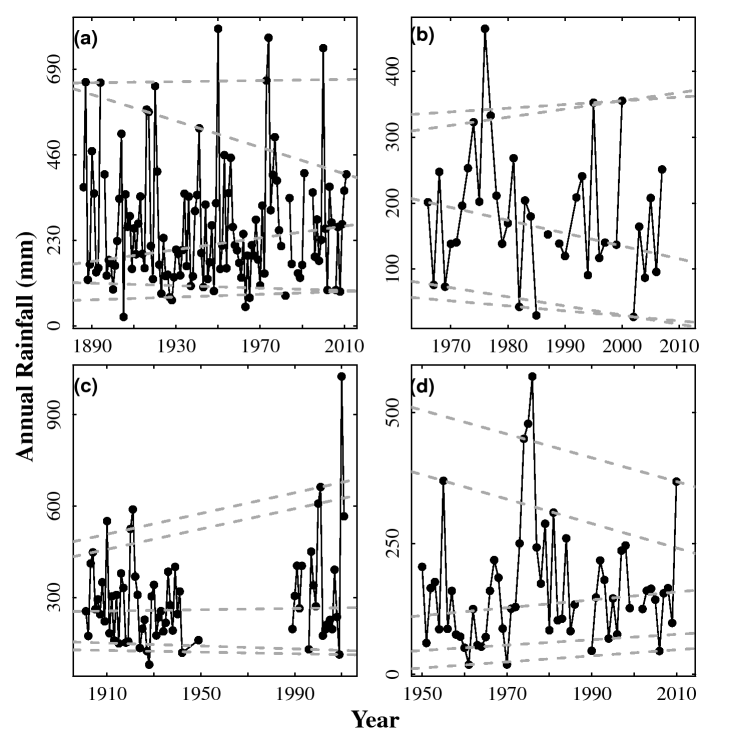

Supplement: Supplementary file 2 [file ece30002-2645-SD2.png]
